# Association of Apolipoprotein E in Lipoprotein Subspecies With Risk of Dementia

**DOI:** 10.1001/jamanetworkopen.2020.9250

**Published:** 2020-07-10

**Authors:** Manja Koch, Steven T. DeKosky, Matthew Goodman, Jiehuan Sun, Jeremy D. Furtado, Annette L. Fitzpatrick, Rachel H. Mackey, Tianxi Cai, Oscar L. Lopez, Lewis H. Kuller, Kenneth J. Mukamal, Majken K. Jensen

**Affiliations:** 1 Department of Nutrition, Harvard T.H. Chan School of Public Health, Boston, Massachusetts; 2 Department of Neurology, University of Florida, Gainesville; 3 Department of Biostatistics, Harvard T.H. Chan School of Public Health, Boston, Massachusetts; 4 Department of Family Medicine, University of Washington, Seattle; 5 Department of Epidemiology, University of Washington, Seattle; 6 Department of Global Health, University of Washington, Seattle; 7 Department of Neurology, University of Pittsburgh, Pittsburgh, Pennsylvania; 8 Department of Epidemiology, University of Pittsburgh Graduate School of Public Health, Pittsburgh, Pennsylvania; 9 Beth Israel Deaconess Medical Center, Department of Medicine, Boston, Massachusetts; 10 Section of Epidemiology, Department of Public Health, University of Copenhagen, Copenhagen, Denmark

## Abstract

**Importance:**

The ε4 allele of the apolipoprotein E (*APOE*) gene and lower apolipoprotein E (apoE) protein levels in plasma are risk factors for Alzheimer disease, but the underlying biological mechanisms are not fully understood. Half of plasma apoE circulates on high-density lipoproteins (HDLs). Higher apoE levels in plasma HDL were previously found to be associated with lower coronary heart disease risk, but the coexistence of another apolipoprotein, apoC3, modified this lower risk.

**Objective:**

To investigate associations between the presence of apoE in different lipoproteins with cognitive function, particularly the risk of dementia.

**Design, Setting, and Participants:**

This prospective case-cohort study embedded in the Ginkgo Evaluation of Memory Study (2000-2008) analyzed data from 1351 community-dwelling participants 74 years and older. Of this group, 995 participants were free of dementia at baseline (recruited from September 2000 to June 2002) and 521 participants were diagnosed with incident dementia during follow-up until 2008. Data analysis was performed from January 2018 to December 2019.

**Exposures:**

Enzyme-linked immunosorbent assay–measured concentration of apoE in whole plasma, HDL-depleted plasma (non-HDL), HDL, and HDL subspecies that contain or lack apoC3 or apoJ.

**Main Outcomes and Measures:**

Adjusted hazard ratios for risk of dementia and Alzheimer disease during follow-up and adjusted differences (β coefficients) in Alzheimer Disease Assessment–Cognitive Subscale (ADAS-cog) and Modified Mini-Mental State Examination scores at baseline.

**Results:**

Among 1351 participants, the median (interquartile range) age was 78 (76-81) years; 639 (47.3%) were women. The median (interquartile range) follow-up time was 5.9 (3.7-6.5) years. Higher whole plasma apoE levels and higher apoE levels in HDL were associated with better cognitive function assessed by ADAS-cog (whole plasma, β coefficient, −0.15; 95% CI, −0.24 to −0.06; HDL, β coefficient, −0.20; 95% CI, −0.30 to −0.10) but were unassociated with dementia or Alzheimer disease risk. When separated by apoC3, a higher apoE level in HDL that lacks apoC3 was associated with better cognitive function (ADAS-cog per SD: β coefficient, 0.17; 95% CI, −0.27 to −0.07; Modified Mini-Mental State Examination score per SD: β coefficient, 0.25; 95% CI, 0.07 to 0.42) and lower risk of dementia (hazard ratio per SD, 0.86; 95% CI, 0.76 to 0.99). In contrast, apoE levels in HDL that contains apoC3 were unassociated with any of these outcomes.

**Conclusions and Relevance:**

In a prospective cohort of older adults with rigorous follow-up of dementia, the apoE level in HDL that lacked apoC3 was associated with better cognitive function and lower dementia risk. This finding suggests that the cardioprotective associations of this novel lipoprotein extend to dementia.

## Key Points

**Question:**

What is the association of apolipoprotein E (apoE) protein levels in different lipoproteins with cognitive function and risk of dementia?

**Findings:**

In this case-cohort study including 1351 community-dwelling participants 74 years and older, the presence of apoE in high-density lipoproteins that lack apoC3 was associated with better cognitive function and decreased risk of dementia. In contrast, the presence of apoE in high-density lipoproteins that contain apoC3 was unrelated to cognitive function and risk of dementia.

**Meaning:**

The findings of this study extend the beneficial associations of the novel apoE–positive, apoC3–negative lipoprotein from cardiovascular disease to dementia.

## Teaser

To evaluate associations of apolipoprotein E protein levels in different lipoproteins with cognitive function and risk of dementia, this case-cohort study embedded in the Ginkgo Evaluation of Memory Study analyzed data from 1351 community-dwelling participants 74 years and older, and found the presence of apoE in high-density lipoproteins that lack apolipoprotein C3 was associated with better cognitive function and decreased risk of dementia.

## Introduction

The ε4 allele of the apolipoprotein E (*APOE*) gene is the most important known single genetic risk factor for late-onset Alzheimer disease (AD),^1,2^ although the mechanism by which it increases AD risk remains incompletely understood. The *APOE* genotype influences plasma apolipoprotein E (apoE) concentrations. Plasma concentrations of apoE themselves are associated with lower risk of dementia and AD, even after accounting for the *APOE* genotype.^3^

Although apoE is a multifunctional protein, research on apoE has focused primarily on its role in lipid binding, transport, and metabolism. It has a major effect on the clearance of plasma lipids by mediating the binding of apoE–containing lipoproteins and lipid complexes to cell-surface lipoprotein receptors.^4,5,6^ In plasma, most apoE is derived from the liver and a constituent of diverse lipoproteins, including very low-density lipoproteins, intermediate-density lipoproteins, chylomicrons, and high-density lipoproteins (HDLs). Given the distinct metabolic roles of these lipoproteins, the association of apoE with dementia might depend on the lipoprotein where it is located. Thus far, investigations of total apoE levels in relation to dementia risk have not accounted for the distribution of apoE among a heterogeneous mix of lipoproteins.

In individuals with normal lipid levels, approximately half of total apoE is found on HDL.^7,8^ Beyond apoE, HDL particles also contain dozens of other proteins.^9^ Thus, HDL can be separated into subfractions based on its protein components.^10^ Accumulating evidence suggests that these subfractions are differentially associated with coronary heart disease (CHD)^11^ and diabetes,^12^ 2 risk factors of dementia.^13,14^ For instance, among generally healthy middle-aged adults free of CHD, higher apoE in HDL is inversely associated with the risk of acute CHD but only in the absence of apoC3 on HDL particles.^15^ These findings support the hypothesis that the properties of plasma lipoproteins and their role in health and disease depend on apolipoprotein composition. Another apolipoprotein found on HDL, apoJ, has been implicated in the pathophysiology of dementia.^16^ The presence of specific apolipoproteins may identify apoE subspecies that are more or less involved in certain disease processes. To address the association of apoE (overall and its subspecies in the fraction of plasma without HDL [non-HDL], in HDL, and in HDL that contains or lacks apoC3 or apoJ) with cognitive function, incidence of dementia, and AD, this study measured the apoE concentration in these lipoprotein subspecies in a prospective, well-phenotyped population of older adults. We hypothesized that higher apoE levels in HDL are inversely associated with risk of dementia but only in the absence of apoC3.

## Methods

### Study Population and Design

The Ginkgo Evaluation of Memory Study (GEMS)^17^ enrolled 3069 community-dwelling participants 74 years and older with normal cognition or mild cognitive impairment recruited from October 2000 to May 2002 at 4 field centers in the US. In the trial,^18^ an intervention of 240 mg of *Ginkgo biloba* daily was found to be ineffective in reducing the incidence of all-cause dementia, but the trial provided an extraordinary resource for secondary analyses because of its dedication of resources to neurologist-adjudicated risk of all-cause dementia and AD.^19^ For the present analysis, we used the case-cohort design, as described Koch et al.^20^ From the 3069 GEMS participants, we included a random subcohort of 1000 participants (32.6%) free of dementia at baseline and an additional 523 (17.0%) participants diagnosed with dementia during follow-up, of whom 166 overlapped (a feature of the case-cohort design) (eFigure 1 in the Supplement). Thus, a total of 1357 participants (44.2% of all GEMS participants) were included in the case-cohort. We excluded 2 participants with no available plasma samples and 4 participants with missing apoE measurements, leaving 1351 participants in the final analysis. Institutional review boards at each investigational center approved the study, and participants and their proxies provided written informed consent. The study meets the criteria for exemption by the Harvard T.H. Chan School of Public Health Office of Human Research Administration per the regulations found at 45 CFR 46.104(d) (4). This study follows the Strengthening the Reporting of Observational Studies in Epidemiology (STROBE) reporting guideline.

### Biochemical Measurements

The nutritional biomarker laboratory at the Harvard T.H. Chan School of Public Health, Boston, Massachusetts, measured the levels of plasma apoE concentrations in different lipoproteins (eFigure 2 in the Supplement) in all participants of this case-cohort study at study entry between September 2000 and June 2002 and for a subset of participants during follow-up (806 participants at 3-year follow-up, 103 participants at 4-year follow-up). To minimize between-plate variation, plasma samples collected from the subset of participants at baseline and follow-up were incubated separately but on the same plates. A total of 307 of the 1351 case-cohort participants (22.7%) self-reported fasting for at least 4 hours at the time of blood sample collection.

We first quantified the concentrations of apoE in whole plasma using a sandwich enzyme-linked immunosorbent assay (ELISA) (Academy Bio-Medical Co). Then, apo B–containing lipoproteins were removed through precipitation using magnesium chloride and dextran sulfate. The concentration of apoE was measured in the remaining fraction (ie, HDL). The concentration of apoE in HDL was subtracted from the concentration of apoE in whole plasma to obtain the concentration of apoE in non-HDL. The measurement of the level of apoE in HDL that contained or lacked apoC3 used a patented and modified sandwich ELISA-based procedure.^9,21^ Antibodies specific for apoC3 (Academy Bio-Medical Co) were precoated onto ELISA plates, and diluted HDL samples were added. After incubation, plates were washed and a tween-containing diluent (1× phosphate-buffered saline with 2% and bovine serum albumin with 0.05% Tween 20) was added to release the lipoproteins that bound to the anti–apoC3 antibodies. Next, these lipoproteins were transferred to a plate precoated with anti–apoE antibodies to quantify the concentration of apoE in HDL that contained apoC3. The concentration of apoE in HDL that contained apoC3 was subtracted from the concentration of apoE in HDL to calculate the concentration of apoE in HDL lacking apoC3. The concentration of apoE in HDL containing or lacking apoJ was measured using the same protocol using apoJ antibodies (R&D Systems). Enzymatic assays were used to measure plasma triglycerides (Thermo Fisher Scientific).

### Dementia Diagnosis and Cognitive Assessment

From 2000 to 2008, participants were assessed semiannually for incident dementia using criteria of the *Diagnostic and Statistical Manual of Mental Disorders* (Fourth Edition). To qualify for study entry, participants underwent a detailed neuropsychological battery of 10 tests capturing the cognitive domains of construction, memory, language, executive function, attention and psychomotor speed, and premorbid intellectual functioning. Participants completed the Clinical Dementia Rating scale and the Modified Mini-Mental State Examination (3MSE) semiannually. From September 2000 to August 2004, the cognitive subscale of the Alzheimer Disease Assessment Scale (ADAS-cog) was administered semiannually, and the results of the 3 cognitive tests were used to mandate readministration of the neuropsychological battery when the scores declined by a prespecified number.^19^ From August 2004 to April 2008, the ADAS-cog and the neuropsychological battery were both administered annually.^22^

Participants considered to potentially have cognitive impairment were referred for a full neurological evaluation and brain magnetic resonance imaging. After this evaluation, a panel reviewed these data and applied a validated protocol for dementia diagnosis (using criteria from the National Institute of Neurological Disorders and Stroke–Association Internationale pour la Recherche et l’Enseignement en Neurosciences, the National Institute of Neurological and Communication Disorders and Stroke, Alzheimer Disease and Related Disorders Association, and the Alzheimer Disease Diagnostic and Treatment Centers).^23,24,25^ Using these criteria, the panel assigned participants with dementia to 1 of the following dementia subtypes: vascular dementia, AD, mixed dementia, or other dementia.^19^ Based on the criteria from the International Working Group on Mild Cognitive Impairment,^26^ participants were considered to have mild cognitive impairment if participants scored at or below the 10th percentile for education-adjusted and age-adjusted norms on 2 or more of 10 selected neuropsychological test scores from each cognitive domain (using the Cardiovascular Health Study population as the norm) and if participants had a score of 0.5 on the Clinical Dementia Rating scale.^27^

### Other Covariates

At study entry, trained technicians collected data on age, sex, educational attainment, smoking status, medical history, and race/ethnicity in interviews, and measured blood pressure, height, and weight. Participants brought prescription drugs and over-the-counter medications to the study visit for entry into the database. Participants were screened for depression using the Center for Epidemiologic Studies-Depression scale. One missing value on this scale was replaced with the population’s median value of 3.

### Statistical Analysis

We recalibrated apoE measures using procedures proposed by Rosner et al^28^ to account for batch-to-batch variation. Among participants with repeated apoE measurements, we assessed the correlation of apoE measured at baseline and follow-up, controlling for sex and age at study entry. The Wilcoxon signed rank test was used to test for equality of apoE and apoE subspecies assessed at study entry and follow-up. To account for the oversampling of participants with dementia in association analyses, participants without dementia were assigned a weight inversely proportional to the sampling probability (3069/1000).

For incident dementia (the primary outcome) and AD, we fit inverse-sampling probability-weighted Cox proportional hazards models with robust estimate of variance, with study time as the underlying time axis censoring at death, drop-out, or dementia diagnosis, whichever occurred first. We tested the proportional hazards assumption based on Schoenfeld residuals.

For cognitive test scores, inverse-sampling probability-weighted linear regression models were used to assess the association of apolipoproteins with the ADAS-cog and 3MSE scores. Because ADAS-cog scores decreased (ie, improved, suggesting learning effects) during the first half of follow-up and showed a nonlinear increase thereafter (eFigure 3 in the Supplement) and because cognitive testing was discontinued in participants diagnosed with dementia during follow-up, we restricted analyses of cognitive scores to baseline values.

The concentration of apoE in whole plasma, HDL-depleted plasma (non-HDL), HDL, and HDL subspecies that contain or lack apoC3 or apoJ were modeled continuously (per 1 standard deviation increment). We adjusted for age, sex, race/ethnicity (as white or nonwhite), clinic site, fasting status (ie, <4 hours or ≥4 hours), education, the weekly number of alcoholic drinks (none, 0.1-0.9, 1.0-7.0, 7.1-14.0, >14, or missing), smoking status (never, former, current, or missing), body mass index (calculated as weight in kilograms divided by height in meters squared; <20, 20-24.9, 25-29.9, ≥30, or missing), lipid-lowering medication use, history of cardiovascular disease, history of diabetes, Center for Epidemiologic Studies-Depression score, treatment assignment from the original trial (placebo or *Ginkgo biloba*), and *APO E* ε4 carrier status (carrier, noncarrier, or missing). Complementary apoE subspecies (eg, apoE in HDL that contained or lacked apoC3) were modeled simultaneously, and likelihood ratio tests were used to assess heterogeneity of the slopes. We conducted sensitivity analyses without adjustment for *APO E* ε4 status and in a subset after exclusion of participants carrying at least 1 *APO E* ε4 allele. Furthermore, in sensitivity analyses, we evaluated the association of the concentration of apoE in HDL that contained or lacked apoC3 with dementia risk after excluding the first 2 years of follow-up. Analyses were performed from January 2018 to December 2019, and used Stata version 12.1 (Stata Corp). Statistical significance was set at 2-sided *P* < .05.

## Results

Among 1351 participants, the median (interquartile range) age was 78 (76-81) years; 639 (47.3%) were women; and 521 participants (38.6%) were diagnosed with dementia, including 352 participants (26.1%) with AD. The median (interquartile range) follow-up time was 5.9 (3.7-6.5) years. Compared with subcohort members, more participants who subsequently developed dementia during follow-up had MCI and carried the *APOE* ε4 allele (dementia cases during follow-up: 197 of 521 participants [38%]; random subcohort: 154 of 995 participants [15.5%]) (Table 1). ApoE and apoE subspecies at baseline and follow-up were highly correlated (all *r* ≥ 0.55) (eFigure 4 in the Supplement). The concentration of apoE in whole plasma and subspecies of apoE in different lipoproteins (apoE concentration in the non-HDL fraction of plasma, in HDL, and in HDL that contains or lacks apoC3 or apoJ) by *APO E* genotype is shown in eTable 1 in the Supplement. There were inverse associations of both apoE in whole plasma and apoE in HDL with *APOE* genotype. Compared with *APOE* ε2/2 and *APO E* ε2/3 carriers, the remaining genotypes had a lower proportion of their total apoE in the HDL fraction of plasma (eg, *APOE* ε2/2, 61% [5th-95th percentile, 35%-95%]; *APOE* ε2/3, 62% [40%-92%]; *APOE* ε3/3, 55% [35%-92%]). Across all genotypes, the HDL fractions containing or lacking apoC3 each contained similar amounts of apoE (Figure). For example, among *APOE* ε2/3 carriers, the concentration of apoE in HDL that contains apoC3 was 5.0 mg/dL (to convert measures apolipoprotein concentration to g/L, multiply mg/dL by 0.01) and the concentration of apoE in HDL that lacks apoC3 was 4.9 mg/dL.

**Table 1.** Baseline Participant Characteristics

Characteristic^a^	Median (5th percentile-95th percentile)	
	Random subcohort (n = 995)	Dementia cases during follow-up (n = 521)
Men, No. (%)	534 (53.7)	265 (50.9)
Age, y	78 (75-85)	79 (75-87)
White, No. (%)	951 (95.6)	489 (93.9)
Education, y	14 (10-20)	14 (8-20)
No. of alcoholic drinks/wk^b^	0.04 (0-16)	0.02 (0-15)
Current smoking, No. (%)^c^	40 (4.0)	20 (3.8)
Body mass index^d^	27 (21-35)	26 (21-34)
Taking lipid-lowering medication, No. (%)	267 (26.8)	160 (30.7)
History of cardiovascular disease, No. (%)	334 (33.6)	197 (37.8)
History of diabetes, No. (%)	87 (8.7)	49 (9.4)
Mild cognitive impairment, No. (%)^e^	154 (15.5)	198 (38.0)
3MSE score at screening visit	94 (85-99)	91 (81-98)
ADAS-cog baseline score	6 (3-11)	8 (4-14)
CES-D scale score	3 (0-10)	4 (0-12)
*Ginkgo biloba* assignment, No. (%)	496 (49.9)	276 (53.0)
*APO E* ε4 allele carrier, No. (%)^f^	180 (18.1)	144 (27.6)
ApoE and apoE subspecies, mg/dL		
Whole plasma apoE	10.8 (6.3-19.2)	10.5 (6.1-18.7)
Non-HDL apoE	4.5 (0.9-9.3)	4.6 (1.0-8.9)
HDL apoE	6.1 (2.7-13.6)	5.9 (2.4-12.4)
ApoE in HDL that contains apoC3	3.2 (1.3-7.1)	3.2 (1.2-6.8)
ApoE in HDL that lacks apoC3	2.8 (0.1-7.8)	2.6 (0.1-6.9)
ApoE in HDL that contains apoJ	0.3 (0.1-0.6)	0.3 (0.1-0.5)
ApoE in HDL that lacks apoJ	5.7 (2.4-13.3)	5.6 (2.1-12.0)
Triglycerides^g^	121 (55-281)	111 (52-251)
All-cause dementia, No. (%)^h^	165 (16.6)	521 (100)
AD	112 (11.3)	352 (67.6)
Vascular dementia	9 (0.9)	24 (4.6)
Mixed dementia	39 (3.9	123 (23.6)
Other dementia	5 (0.5)	22 (4.2)

Abbreviations: 3MSE, Modified Mini-Mental State Examination score; AD, Alzheimer dementia; ADAS-cog, Alzheimer Disease Assessment Scale Cognitive subscale; apo, apolipoprotein; *APOE*, Apolipoprotein E gene; CES-D, Center for Epidemiologic Studies-Depression scale; HDL, high-density lipoprotein.

SI conversion factors: To convert apo measurements to grams per liter, multiply by 0.01; to convert triglycerides to millimoles per liter, multiply by 0.0113.

^a^ Percentages are calculated with missing data.

^b^ Data from 21 missing.

^c^ Data from 24 missing.

^d^ Data from 7 missing. Body mass index is calculated as weight in kilograms divided by height in meters squared.

^e^ Mild cognitive impairment was diagnosed if participants scored less than or equal to the 10th percentile for age and education on at least 2 tests of the neuropsychological battery using the Cardiovascular Health Study population as a reference population, while also having a Clinical Dementia Rating global score of 0.5.^27^

^f^ Data from 288 missing.

^g^ Data from 6 missing.

^h^ Per the case-cohort study design, the 165 participants who developed dementia within the random subcohort were included in both the case count and the subcohort count.

**Figure.**
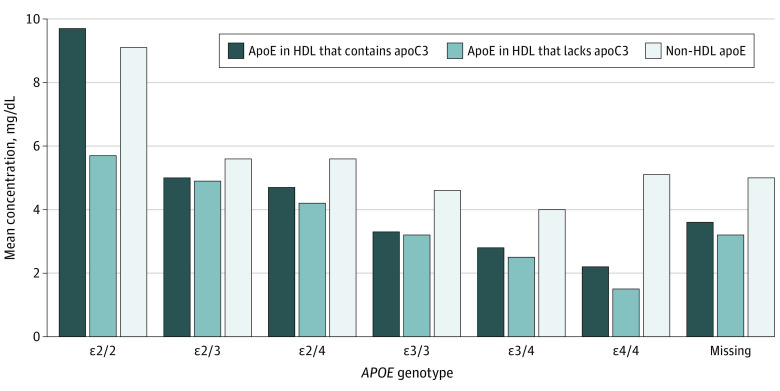
Concentration of Plasma Apolipoprotein E (ApoE) in Different Lipoprotein Subspecies by *APOE* Genotype Mean concentration of non–high-density lipoprotein (HDL) apoE and apoE in HDL containing or lacking apoC3 at screening visit by *APOE* genotype in 995 older individuals selected as a random subcohort of the Ginkgo Evaluation of Memory study.^17^ *APOE* indicates apolipoprotein E gene.

### ApoE in Whole Plasma, Non-HDL, and HDL

Higher apoE levels in whole plasma, in non-HDL, and in HDL were not significantly associated with risk of dementia or AD in fully adjusted models (Table 2; eTable 2 in the Supplement). We assessed associations of lipoprotein species with cognition at baseline (Table 2). Higher apoE in whole plasma and higher apoE levels in HDL (but not in non-HDL) were associated with lower ADAS-cog scores at baseline, indicating better cognitive function (whole plasma, β coefficient, −0.15; 95% CI, −0.24 to −0.06; HDL, β coefficient, −0.20; 95% CI, −0.30 to −0.10). The concentration of apoE in whole plasma, non-HDL, or HDL were not statistically associated with 3MSE scores.

**Table 2.** Hazard Ratios for Risk of Dementia and the Differences in Cognitive Scores at Baseline According to Concentrations of ApoE in Whole Plasma, Non-HDL Plasma, and HDL Plasma at Screening Visit in 1351 Participants of the Ginkgo Evaluation of Memory Case-Cohort

Concentration of apoE	Mean (SD), mg/dL	Hazard ratio per 1-SD increase (95% CI)		Difference in cognitive scores per 1-SD increase, β (95% CI)	
		Dementia^a^	AD^a^	ADAS-cog	3MSE
Whole plasma	4.4	0.92 (0.82 to 1.03)	0.88 (0.77 to 1.00)	–0.15 (–0.24 to –0.06)	0.12 (–0.03 to 0.28)
Non-HDL	2.8	0.89 (0.77 to 1.03)	0.88 (0.75 to 1.03)	–0.06 (–0.17 to 0.05)	0.14 (–0.05 to 0.33)
HDL	3.5	0.94 (0.83 to 1.07)	0.88 (0.75 to 1.02)	–0.20 (–0.30 to –0.10)	0.10 (–0.07 to 0.27)

Abbreviations: AD, Alzheimer disease; ADAS-cog, Alzheimer Disease Assessment Scale Cognitive subscale; apo, apolipoprotein; *APOE*, Apolipoprotein E gene; HDL, high-density lipoprotein; 3MSE, Modified Mini-Mental State Examination score.

SI conversion factors: To convert apo measurements to grams per liter, multiply by 0.01.

^a^ Hazard ratios were obtained from weighted Cox proportional hazard regression models, and differences in cognitive scores were obtained from weighted linear regression models adjusted for age, sex, race/ethnicity, clinic site, fasting status, education, weekly number of alcoholic drinks, smoking status, body mass index, lipid-lowering medication use, history of cardiovascular disease, history of diabetes, Center for Epidemiologic Studies Depression scale score, treatment assignment, and *APOE* ε4 carrier status. The concentration apoE in whole plasma, non-HDL, and HDL was modeled separately.

### ApoE in HDL Containing or Lacking ApoC3 or ApoJ

In contrast with total apoE, a higher apoE level in HDL that lacked apoC3 was significantly associated with lower dementia risk (Table 3). The hazard ratios (HRs) for apoE level in HDL that contained or lacked apoC3 with dementia risk were statistically significantly different (HR per 1-SD higher apoE in HDL that contained apoC3: 1.07 [95% CI, 0.95-1.19] vs 0.86 [95% CI, 0.76-0.99]; *P* for heterogeneity = .03). ApoE subspecies had similar associations with AD as with all-cause dementia. In sensitivity analyses excluding the first 2 years of follow-up, the results were not materially different. The HR for dementia per 1-SD higher concentration of apoE in HDL that contained apoC3 was 1.11 (95% CI, 0.98-1.25) and was 0.87 (95% CI, 0.76-1.00) for apoE in HDL that lacked apoC3.

**Table 3.** Hazard Ratios for Risk of Dementia and the Difference in Cognitive Scores at Baseline According to Concentrations of ApoE in HDL That Contains or Lacks ApoC3 at Screening Visit in 1351 Participants of the Ginkgo Evaluation of Memory Case-Cohort

Characteristic	SD, mg/dL	Hazard ratio per 1-SD increase (95% CI)^a^		Difference in cognitive scores per 1-SD increase, β (95% CI)	
		Dementia	AD	ADAS-cog	3MSE
ApoE in HDL that contains apoC3	2.0	1.07 (0.95 to 1.19)	1.00 (0.87 to 1.14)	–0.08 (–0.17 to 0.01)	–0.12 (–0.28 to 0.04)
ApoE in HDL that lacks apoC3	2.4	0.86 (0.76 to 0.99)	0.86 (0.73 to 1.00)	–0.17 (–0.27 to –0.07)	0.25 (0.07 to 0.42)

Abbreviations: AD, Alzheimer disease; ADAS-cog, Alzheimer Disease Assessment Scale Cognitive subscale; apo, apolipoprotein; *APOE*, Apolipoprotein E gene; HDL, high-density lipoprotein; 3MSE, Modified Mini-Mental State Examination.

To convert apo measures to g/L, multiply mg/dL by 0.01.

^a^ Hazard ratios were obtained from weighted Cox proportional hazard regression models, and differences in the cognitive scores were obtained from weighted linear regression models adjusted for age, sex, race/ethnicity, clinic site, fasting status, education, weekly number of alcoholic drinks, smoking status, body mass index, lipid-lowering medication use, history of cardiovascular disease, history of diabetes, Center for Epidemiologic Studies-Depression scale score, treatment assignment, and *APOE* ε4 carrier status (carrier, noncarrier, missing). The apoE level in HDL containing apoC3 and the apoE level in HDL lacking apoC3 were included as continuous variables simultaneously in models.

A higher apoE level in HDL that lacked apoC3 was also associated with lower ADAS-cog scores (ie, better cognitive function) at baseline, while a higher apoE level in HDL that contained apoC3 was not associated with ADAS-cog scores (difference in ADAS-cog score per 1-SD higher apoE in HDL that contains apoC3: −0.08 [95% CI, −0.17 to 0.01]; difference in ADAS-cog score per 1-SD higher apoE in HDL that lacks apoC3: −0.17 [95% CI, −0.27 to −0.07]) (Table 3). Furthermore, the concentration of apoE in HDL containing or lacking apoJ was differentially associated with ADAS-cog scores (difference in ADAS-cog score per 1-SD higher apoE in HDL that contains apoJ, 0.05 [95% CI, −0.06 to −0.16]; difference in ADAS-cog score per 1-SD higher apoE in HDL that lacks apoJ, −0.24 [95% CI, −0.36 to −0.12]; *P* for heterogeneity = .005) but not with dementia (Table 4). Consistent with the ADAS-cog results, apoE levels in HDL containing or lacking apoC3 as well as apoE levels in HDL containing or lacking apoJ were differentially associated with 3MSE scores. A higher apoE level in HDL that lacked apoC3 was associated with higher 3MSE scores (ie, better cognitive function) at baseline (difference in 3MSE score per 1-SD higher apoE in HDL that contains apoC3, −0.12 [95% CI, −0.28 to 0.04]; difference in 3MSE score per 1-SD higher apoE in HDL that lacks apoC3, 0.25 [95% CI, 0.07 to 0.42]; *P* for heterogeneity = .005), while a higher apoE level in HDL that contained apoC3 was unassociated with 3MSE scores. Higher apoE levels in HDL containing apoJ were associated with lower 3MSE scores (difference in 3MSE score per 1-SD higher apoE in HDL that contains apoJ, −0.22 [95% CI, −0.41 to −0.02]; *P* for heterogeneity = .01). In contrast, higher apoE levels in HDL lacking apoJ were associated with higher 3MSE scores (difference in 3MSE score per 1-SD higher apoE in HDL that lacks apoJ, 0.24 [95% CI, 0.03 to 0.44]).

**Table 4.** Hazard Ratios for Risk of Dementia and the Difference in Cognitive Scores at Baseline According to ApoE in HDL That Contains or Lacks ApoJ at Screening Visit in 1351 Participants of the Ginkgo Evaluation of Memory Case-Cohort

Characteristic	SD, mg/dL	Hazard ratios per 1-SD increase (95% CI)^a^		Difference in cognitive scores per 1-SD increase, β (95% CI)	
		Dementia	AD	ADAS-cog	3MSE
ApoE in HDL that contains apoJ	0.1	1.02 (0.88 to 1.17)	0.93 (0.78 to 1.10)	0.05 (–0.06 to 0.16)	–0.22 (–0.41 to –0.02)
ApoE in HDL that lacks apoJ	3.5	0.93 (0.80 to 1.08)	0.92 (0.77 to 1.09)	–0.24 (–0.36 to –0.12)	0.24 (0.03 to 0.44)

Abbreviations: AD, Alzheimer disease; ADAS-cog, Alzheimer Disease Assessment Scale Cognitive subscale; apo, apolipoprotein; *APOE*, Apolipoprotein E gene; HDL, high-density lipoprotein; 3MSE, Modified Mini-Mental State Examination score.

To convert apolipoprotein measures to g/L, multiply mg/dL by 0.01.

^a^ Hazard ratios were obtained from weighted Cox proportional hazard regression models, and differences in the cognitive scores were obtained from weighted linear regression models adjusted for age, sex, race/ethnicity, clinic site, fasting status, education, weekly number of alcoholic drinks, smoking status, body mass index, lipid-lowering medication use, history of cardiovascular disease, history of diabetes, Center for Epidemiologic Studies-Depression scale score, treatment assignment, and *APOE* ε4 carrier status (carrier, noncarrier, missing). The apoE level in HDL containing apoJ and the apoE level in HDL lacking apoJ were included as continuous variables simultaneously in models.

## Discussion

In this study analyzing the association of apoE level in HDL with cognition and dementia, substantial differences existed based on the presence or absence of other apolipoproteins in the HDL fractions. Specifically, the presence of apoC3 in HDL appeared to modulate the association of apoE in HDL with risk of dementia and AD. While the concentration of apoE in HDL that contained apoC3 was not associated with either end point, higher concentrations of apoE in HDL that lacked apoC3 were associated with a lower risk of both total dementia and AD.

Large-scale studies without information on lipoprotein subspecies have demonstrated that higher plasma apoE is associated with less cognitive decline and a lower risk of subsequent AD and other dementias.^29,30^ Consistent with these studies, we found an inverse association between apoE in HDL and dementia and AD risk in basic adjusted models. However, the association did not persist after adjustment for lifestyle factors and clinical characteristics. It is possible that apoE levels are less strongly associated with dementia risk in older populations like that studied in the GEMS.^17^ On the other hand, the association observed in other studies may reflect an unrecognized association restricted to apoE in HDL without apoC3.

Although apoE is expressed in the brain, the major source of apoE in plasma is the liver, and plasma and brain pools are distinct^31^ but correlated in some but not all studies.^32,33^ So far, most studies have focused on neuropathological effects of the apoE ε4 isoform within the central nervous system, while mechanisms by which circulatory apoE might contribute to cognitive decline and onset of AD and AD pathology are poorly understood.^34^ The observation that apoE deficiency in mice is associated with blood–brain barrier dysfunction has led to the hypothesis that apoE is involved in cerebrovascular integrity.^35^

With regard to apoE subspecies, in the absence of apoC3, higher apoE in the HDL fraction of plasma was associated with better cognitive function and a lower risk of dementia and AD. The latter findings parallel observations from the Danish Diet, Cancer, and Health study,^15^ in which higher apoE in plasma HDL was associated with lower CHD risk,^15^ but as this study found, only in the absence of apoC3. Lower CHD risk is itself a risk factor for dementia. Although it is not known if apoE in HDL without apoC3 is causally associated with lower risk of dementia or cardiovascular disease, metabolic studies have shown that while HDL that contains apoE is cleared from the circulation more than 7-fold faster than HDL that lacks apoE, the clearance of HDL is attenuated if apoE coexists with apoC3.^15^ Thus, at least for clearance, apoC3 effectively counteracts the effect of apoE—similar to what we have now observed for both CHD and dementia.

Despite the strong genetic association of *APOJ* with dementia, population-based studies linking plasma apoJ levels to AD risk have provided inconsistent results.^36,37^ The high affinity of apoJ to bind amyloid β in cerebrospinal fluid has led to suggestions that apoJ has a role in AD pathology.^38^ In vitro studies demonstrate that amyloid β–apoJ complexes bind the low-density lipoprotein receptor–related protein 2, also called megalin, resulting in amyloid β clearance from the brain.^39^ Although apoE can also bind to megalin, the functional role of this interaction is incompletely understood.^40^ The A variant of the megalin promotor polymorphism rs3755166 leads to lower transcript levels of the megalin protein and has been associated with higher risk of AD and greater cognitive decline in prior studies.^41,42,43^

Studies addressing potential interactions of plasma apolipoproteins on HDL are sparse. Apo A1 is the main structural apolipoprotein found in all circulatory HDL. In 1 of our previous investigations among GEMS participants,^21^ the amount of apoA1 in HDL was not associated with cognitive function or risk of dementia, regardless of the presence of apoC3, apoJ, or apoE in HDL. However, in a substudy of GEMS participants who remained free of clinical dementia, the amount of apoE in subspecies of HDL that contained or lacked apoC3 or apoJ was uniformly inversely associated with amyloid β deposition on positron emission tomographic scans.^34^ Thus, the amount of apoE, rather than the amount of apoA1, might be particularly strongly associated with dementia risk. Further studies are needed to clarify if the amount of apoE in HDL containing or lacking apoC3 or apoJ is associated with dementia via brain-specific or systemic pathways.

### Limitations and Strengths

Important limitations warrant mention. Our study population was limited to older participants, and the underlying biological processes leading to incident dementia during follow-up were presumably already underway, thus making generalization to younger populations difficult. Cognitive testing was systematic but was truncated when dementia (the primary end point of GEMS^17^) occurred because it served primarily as a method to trigger formal dementia ascertainment and adjudication. Mild cognitive impairment was not a trial outcome and could not be classified retrospectively because, up until 2004, the full neuropsychological battery was administered only to participants with scores of the brief battery declining by a prespecified number.

This study also had strengths. To our knowledge, this study is the first examination of novel apoE subspecies. Other major strengths of this study are the detailed, frequent, and prospective evaluation of neurological status, the substantial number of participants with dementia, and the comprehensive data available for adjustment, including by *APOE* genotype.

## Conclusions

In this study, higher apoE levels in HDL lacking apoC3 in an older population were associated with better cognitive function and a lower risk of dementia or AD. In contrast, apoE in HDL that contained apoC3 was unassociated with cognitive function and risk of dementia or AD. The study suggests that previously identified cardioprotective associations of this novel apolipoprotein extend to dementia and highlights the need for studies that assess the relevance of apoE in HDL that lacks apoC3 as new targets for modifiable behavioral or therapeutic interventions and risk stratification.

## Notes

Supplement. **eTable 1.** ApoE and ApoE Subspecies at Screening Visit by *APOE* Genotype in the Random Subcohort of the Ginkgo Evaluation of Memory Study (N=995) **eTable 2.** Hazard Ratios (HRs) for Risk of Dementia or Difference in Cognitive Scores at Baseline and 95% Confidence Intervals (95% CI) per SD Higher Plasma Concentrations of ApoE and ApoE Subspecies at Screening Visit in the Ginkgo Evaluation of Memory Case-cohort, N=1351 **eFigure 1.** Flowchart Summarizing Case-cohort Sampling **eFigure 2.** Measurement of Plasma Concentration of ApoE in Different Lipoprotein Subspecies by ELISA **eFigure 3.** Average Trajectories of ADAS-cog Scores by Tertiles of Whole Plasma ApoE (A) and ApoE in HDL (B) at Screening Visit in the Ginkgo Evaluation of Memory Case-cohort **eFigure 4.** HDL and its Apolipoprotein-defined Subspecies and Other Apolipoproteins at Study Entry (2000 to 2002) and Follow-up
