# Supplementary material for: Association of Apolipoprotein E in Lipoprotein Subspecies With Risk of Dementia
Source: JAMA Netw Open. 2020 Jul 10;3(7):e209250. doi: 10.1001/jamanetworkopen.2020.9250 (PMC7352155; doi:10.1001/jamanetworkopen.2020.9250)
Supplement: Supplement. — eTable 1. ApoE and ApoE Subspecies at Screening Visit by APOE Genotype in the Random Subcohort of the Ginkgo Evaluation of Memory Study (N=995) eTable 2. Hazard Ratios (HRs) for Risk of Dementia or Difference in Cognitive Scores at Baseline and 95% Confidence Intervals (95% CI) per SD Higher Plasma Concentrations of ApoE and ApoE Subspecies at Screening Visit in the Ginkgo Evaluation of Memory Case-cohort, N=1351 eFigure 1. Flowchart Summarizing Case-cohort Sampling eFigure 2. Measurement of Plasma Concentration of ApoE in Different Lipoprotein Subspecies by ELISA eFigure 3. Average Trajectories of ADAS-cog Scores by Tertiles of Whole Plasma ApoE (A) and ApoE in HDL (B) at Screening Visit in the Ginkgo Evaluation of Memory Case-cohort eFigure 4. HDL and its Apolipoprotein-defined Subspecies and Other Apolipoproteins at Study Entry (2000 to 2002) and Follow-up [file jamanetwopen-3-e209250-s001.pdf]

Supplementary Online Content

Koch M, DeKosky ST, Goodman M, et al. Association of apolipoprotein E in lipoprotein subspecies with risk of dementia. *JAMA Netw Open*. 2020;3(7):e209250. doi:10.1001/jamanetworkopen.2020.9250

eTable 1. ApoE and ApoE Subspecies at Screening Visit by *APOE* Genotype in the Random Subcohort of the Ginkgo Evaluation of Memory Study (N=995)

eTable 2. Hazard Ratios (HRs) for Risk of Dementia or Difference in Cognitive Scores at Baseline and 95% Confidence Intervals (95% CI) per SD Higher Plasma Concentrations of ApoE and ApoE Subspecies at Screening Visit in the Ginkgo Evaluation of Memory Case-cohort, N=1351

eFigure 1. Flowchart Summarizing Case-cohort Sampling

eFigure 2. Measurement of Plasma Concentration of ApoE in Different Lipoprotein Subspecies by ELISA

eFigure 3. Average Trajectories of ADAS-cog Scores by Tertiles of Whole Plasma ApoE (A) and ApoE in HDL (B) at Screening Visit in the Ginkgo Evaluation of Memory Case-cohort

eFigure 4. HDL and its Apolipoprotein-defined Subspecies and Other Apolipoproteins at Study Entry (2000 to 2002) and Follow-up

This supplementary material has been provided by the authors to give readers additional information about their work.

© 2020 Koch M et al. *JAMA Netw Open.*

eTable 1. Concentration of apoE and apoE subspecies[1] at screening visit by *APOE* genotype in the random subcohort of the Ginkgo Evaluation of Memory study (N=995)

	APOE ε2/2 (N=8)	*APOE ε2/3* (N=89)	*APOE ε2/4* (N=19)	*APOE ε3/3* (N=518)	*APOE ε3/4* (N=152)	*APOE ε4/4* (N=9)	*APOE* genotype missing (N=200)
Whole plasma apoE, mg/dl	26.0 (12.8, 34.8)	14.8 (9.2, 23.6)	13.6 (8.0, 25.4)	10.6 (6.5, 17.7)	9.1 (5.7, 14.9)	8.2 (4.8, 14.4)	10.7 (6.3, 20.3)
Non-HDL apoE, mg/dl	8.3 (0.7, 19.4)	5.5 (0.9, 11.3)	5.6 (1.2, 9.6)	4.4 (0.8, 8.8)	3.7 (0.4, 7.9)	5.1 (3.6, 6.6)	4.5 (0.8, 9.1)
HDL apoE, mg/dl	15.6 (8.6, 25.6)	9.2 (5.1, 15.6)	8.2 (3.2, 21.4)	5.9 (2.8, 12.1)	4.8 (2.2, 9.4)	3.3 (1.2, 9.9)	5.8 (2.4, 12.5)
% whole plasma apoE in HDL	61 (35, 95)	62 (40, 92)	55 (39, 89)	55 (35, 92)	56 (31, 94)	39 (22, 69)	54 (33, 93)
ApoE in HDL that contains apoC3, mg/dl	8.1 (5.2, 17.9)	4.8 (2.5, 8.2)	4.1 (2.0, 13.4)	3.1 (1.2, 6.5)	2.6 (0.9, 5.2)	2.0 (0.6, 5.5)	3.2 (1.3, 7.6)
ApoE in HDL that lacks apoC3, mg/dl	6.0 (0.004, 13.8)	4.4 (0.7, 10.3)	3.9 (0.3, 11.6)	2.7 (0.1, 7.2)	2.3 (0.004, 5.7)	0.7 (0.004, 4.5)	2.6 (0.04, 7.5)
ApoE in HDL that contains apoJ, mg/dl	0.6 (0.2, 0.9)	0.3 (0.2, 0.6)	0.3 (0.2, 0.6)	0.3 (0.2, 0.6)	0.3 (0.1, 0.4)	0.3 (0.1, 0.4)	0.3 (0.2, 0.6)
ApoE in HDL that lacks apoJ, mg/dl	14.8 (0.8, 24.9)	8.9 (4.8, 15.2)	7.8 (2.9, 20.8)	5.6 (2.6, 11.8)	4.6 (2.0, 9.1)	3.1 (1.0, 9.5)	5.4 (2.2, 12.1)

Abbreviations: Apo, Apolipoprotein. *APOE*, Apolipoprotein E gene. HDL, High-density lipoprotein.
[1]Values are median (Q5; Q95).

© 2020 Koch M et al. *JAMA Netw Open.*

eTable 2. Hazard ratios (HRs)[1] for risk of dementia or difference in cognitive scores at baseline and 95% confidence intervals (95% CI) per SD higher plasma concentrations of apoE and apoE subspecies at screening visit in the Ginkgo Evaluation of Memory case-cohort, N=1351.

	HR for dementia (95% CI) per SD	HR (95% CI) for AD per SD	Difference in ADAS-cog (95% CI) per SD	Difference in 3MSE (95% CI) per SD
Whole plasma apoE				
Basic	**0.84 (0.75, 0.94)**	**0.79 (0.69, 0.91)**	**-0.21 (-0.30, -0.12)**	**0.20 (0.04, 0.36)**
Multivariable	**0.87 (0.78, 0.98)**	**0.82 (0.71, 0.94)**	**-0.18 (-0.26, -0.09)**	0.14 (-0.01, 0.29)
Multivariable + *APOE*[2]	0.92 (0.82, 1.03)	0.88 (0.77, 1.00)	**-0.15 (-0.24, -0.06)**	0.12 (-0.03, 0.28)
Multivariable excluding *APOE* ε4 carriers[3]	0.91 (0.80, 1.03)	0.86 (0.73, 1.00)	**-0.14 (-0.23, -0.05)**	0.08 (-0.09, 0.24)
Non-HDL apoE				
Basic	**0.85 (0.74, 0.98)**	**0.85 (0.73, 0.99)**	**-0.12 (-0.23, -0.01)**	**0.24 (0.04, 0.44)**
Multivariable	0.88 (0.76, 1.02)	0.87 (0.74, 1.02)	-0.07 (-0.18, 0.04)	0.15 (-0.04, 0.34)
Multivariable + *APOE*[2]	0.89 (0.77, 1.03)	0.88 (0.75, 1.03)	-0.06 (-0.17, 0.05)	0.14 (-0.05, 0.33)
Multivariable excluding *APOE* ε4 carriers[2]	0.89 (0.75, 1.05)	0.87 (0.73, 1.05)	-0.07 (-0.19, 0.04)	**0.23 (0.03, 0.43)**
HDL apoE				
Basic	**0.85 (0.74, 0.96)**	**0.77 (0.65, 0.90)**	**-0.25 (-0.35, -0.15)**	0.16 (-0.02, 0.34)
Multivariable	**0.87 (0.77, 0.99)**	**0.80 (0.68, 0.93)**	**-0.23 (-0.33, -0.13)**	0.12 (-0.05, 0.29)
Multivariable + *APOE*[2]	0.94 (0.83, 1.07)	0.88 (0.75, 1.02)	**-0.20 (-0.30, -0.10)**	0.10 (-0.07, 0.27)
Multivariable excluding *APOE* ε4 carriers[2]	0.92 (0.79, 1.07)	0.85 (0.70, 1.02)	**-0.18 (-0.28, -0.07)**	-0.03 (-0.21, 0.16)
ApoE in HDL that contains apoC3				
Basic	1.02 (0.91, 1.14)	0.92 (0.80, 1.06)	-0.09 (-0.18, 0.003)	-0.10 (-0.26, 0.07)
Multivariable	1.03 (0.92, 1.15)	0.94 (0.82, 1.08)	**-0.10 (-0.19, -0.01)**	-0.11 (-0.26, 0.05)
Multivariable + *APOE*[2]	1.07 (0.95, 1.19)	1.00 (0.87, 1.14)	-0.08 (-0.17, 0.01)	-0.12 (-0.28, 0.04)
Multivariable excluding *APOE* ε4 carriers[2]	1.05 (0.92, 1.20)	0.95 (0.80, 1.13)	-0.04 (-0.14, 0.05)	-0.15 (-0.32, 0.02)
ApoE in HDL that lacks apoC3				
Basic	**0.79 (0.69, 0.91)**	**0.78 (0.66, 0.92)**	**-0.23 (-0.33, -0.13)**	**0.31 (0.12, 0.49)**
Multivariable	**0.82 (0.72, 0.94)**	**0.80 (0.68, 0.94)**	**-0.19 (-0.29, -0.10)**	**0.26 (0.09, 0.44)**
Multivariable + *APOE*[2]	**0.86 (0.76, 0.99)**	0.86 (0.73, 1.00)	**-0.17 (-0.27, -0.07)**	**0.25 (0.07, 0.42)**
Multivariable excluding *APOE* ε4 carriers[2]	0.86 (0.74, 1.00)	0.86 (0.72, 1.03)	**-0.18 (-0.29, -0.07)**	0.12 (-0.07, 0.31)
ApoE in HDL that contains apoJ				
Basic	0.96 (0.84, 1.11)	0.86 (0.73, 1.01)	0.08 (-0.03, 0.19)	**-0.23 (-0.42, -0.03)**
Multivariable	1.00 (0.86, 1.15)	0.90 (0.76, 1.07)	0.05 (-0.06, 0.16)	**-0.22 (-0.41, -0.02)**
Multivariable + *APOE*[2]	1.02 (0.88, 1.17)	0.93 (0.78, 1.10)	0.05 (-0.06, 0.16)	**-0.22 (-0.41, -0.02)**
Multivariable excluding *APOE* ε4 carriers[2]	0.99 (0.84, 1.16)	0.85 (0.69, 1.05)	0.04 (-0.07, 0.16)	**-0.23 (-0.43, -0.02)**

eTable 2 continued on next page

© 2020 Koch M et al. *JAMA Netw Open.*

eTable 2 *continued.* Hazard ratios (HRs)[1] for risk of dementia or difference in cognitive scores at baseline and 95% confidence intervals (95% CI) per SD higher plasma concentrations of apoE and apoE subspecies at screening visit in the Ginkgo Evaluation of Memory case-cohort, N=1351.

	HR for dementia (95% CI) per SD	HR (95% CI) for AD per SD	Difference in ADAS-cog (95% CI) per SD	Difference in 3MSE (95% CI) per SD
ApoE in HDL that lacks apoJ				
Basic	0.86 (0.74, 1.00)	0.83 (0.70, 1.00)	**-0.31 (-0.43, -0.19)**	**0.31 (0.09, 0.52)**
Multivariable	0.87 (0.75, 1.01)	0.84 (0.70, 1.01)	**-0.26 (-0.38, -0.15)**	**0.26 (0.05, 0.47)**
Multivariable + *APOE*[2]	0.93 (0.80, 1.08)	0.92 (0.77, 1.09)	**-0.24 (-0.36, -0.12)**	**0.24 (0.03, 0.44)**
Multivariable excluding *APOE* ε4 carriers[2]	0.93 (0.78, 1.10)	0.92 (0.75, 1.13)	**-0.23 (-0.36, -0.12)**	0.12 (-0.11, 0.34)

Abbreviations: ADAS-cog, Cognitive subscale of the Alzheimer Disease Assessment Scale. Apo, Apolipoprotein. *APOE*, Apolipoprotein E gene. HDL, High-density lipoprotein. 3MSE, Modified Mini-Mental State Examination score.
[1]Hazard Ratios (HRs) were obtained from weighted Cox proportional hazard regression models adjusted for age, sex, race/ethnicity, clinic site, fasting status. Each lipoprotein was modeled separately, except for complementary subspecies (e.g., apoE in HDL containing or lacking apoC3) which were included simultaneously in models. Multivariable analyses were adjusted for age, sex, race/ethnicity, clinic site, fasting status, education, weekly number of alcoholic drinks, smoking status, body mass index, lipid-lowering medication use, history of cardiovascular disease, history of diabetes, CES-D, and treatment assignment. [2]Multivariable analyses were additionally adjusted for *APOE* ε4 carrier status (*APOE ε4* carrier, *APOE ε4* non-carrier, missing). [3]N=1070.

© 2020 Koch M et al. *JAMA Netw Open.*

eFigure 1. Flowchart summarizing case-cohort sampling.

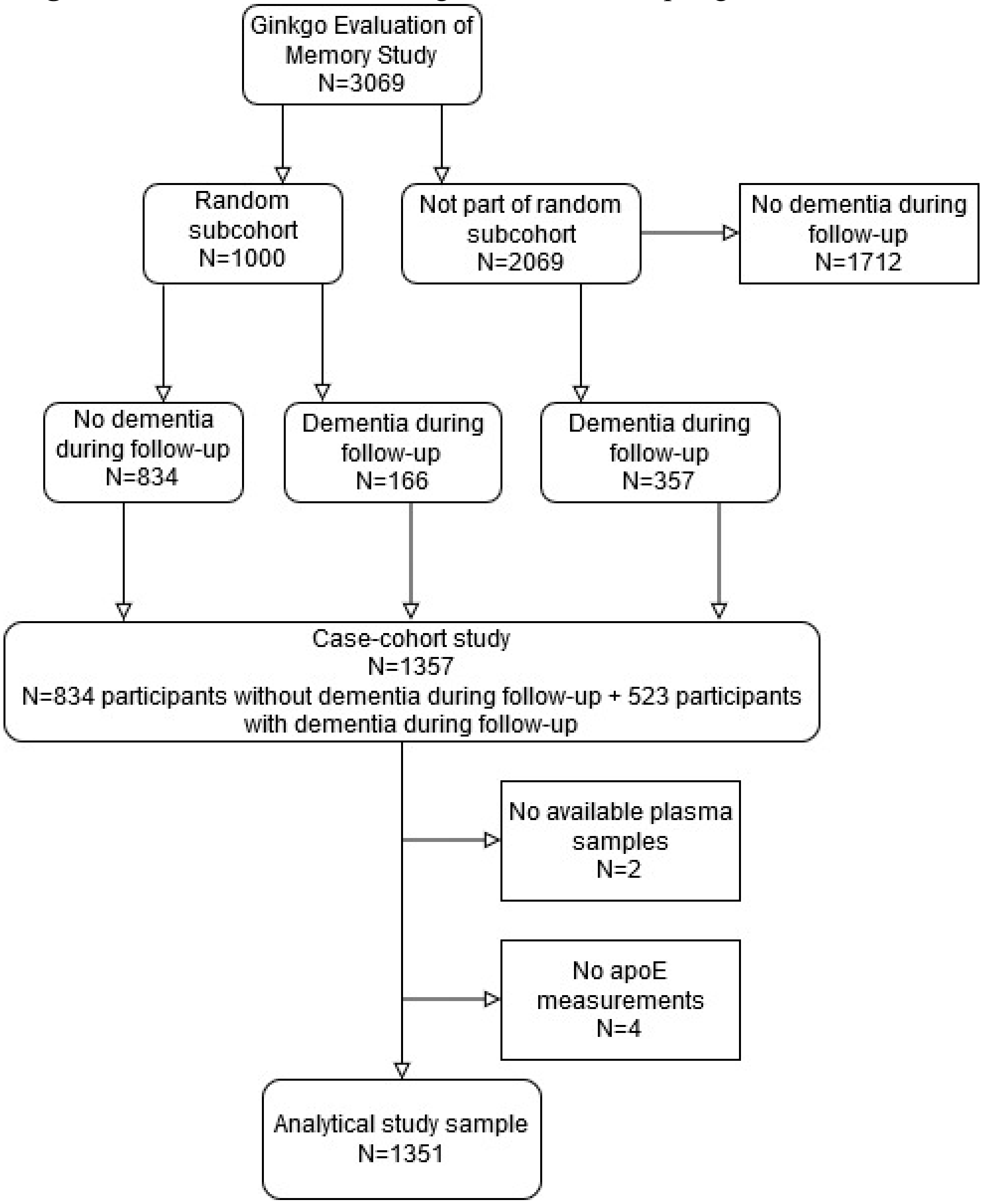

© 2020 Koch M et al. *JAMA Netw Open.*

eFigure 2. Measurement of plasma concentration of apoE in different lipoprotein subspecies by ELISA.

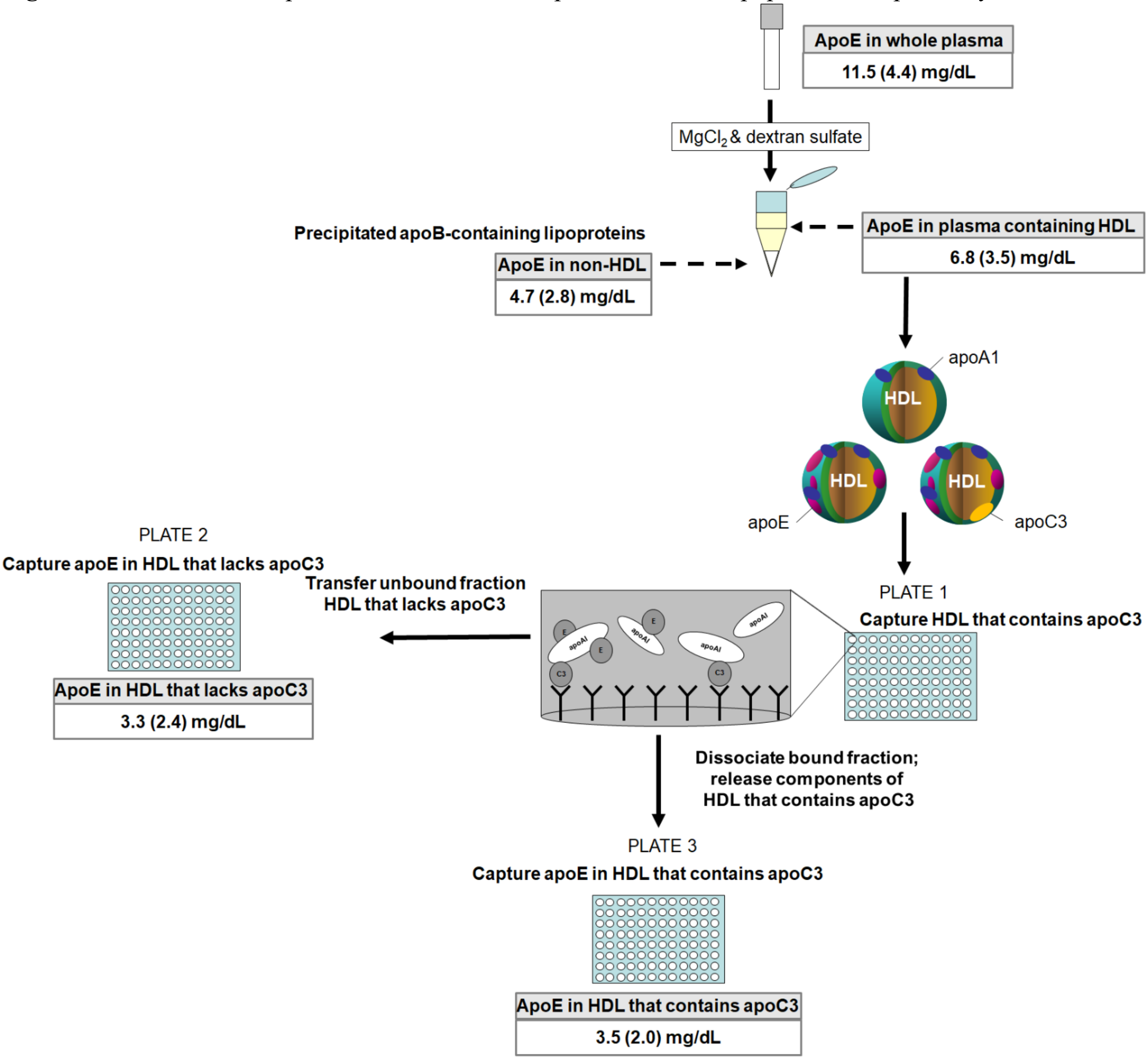

Concentrations (mean [SD]) of apoE in whole plasma, in the fraction of plasma without HDL (non-HDL), in HDL, and in HDL that contains or lacks apoC3 refer to the random subcohort of the Ginkgo Evaluation of Memory study (N=995).

Abbreviations: Apo, Apolipoprotein. ELISA, Enzyme-linked immunosorbent assay. HDL, High-density lipoprotein.

© 2020 Koch M et al. *JAMA Netw Open.*

eFigure 3. Average trajectories of ADAS-cog scores by tertiles of whole plasma apoE (A) and apoE in HDL (B) at screening visit in the Ginkgo Evaluation of Memory case-cohort.

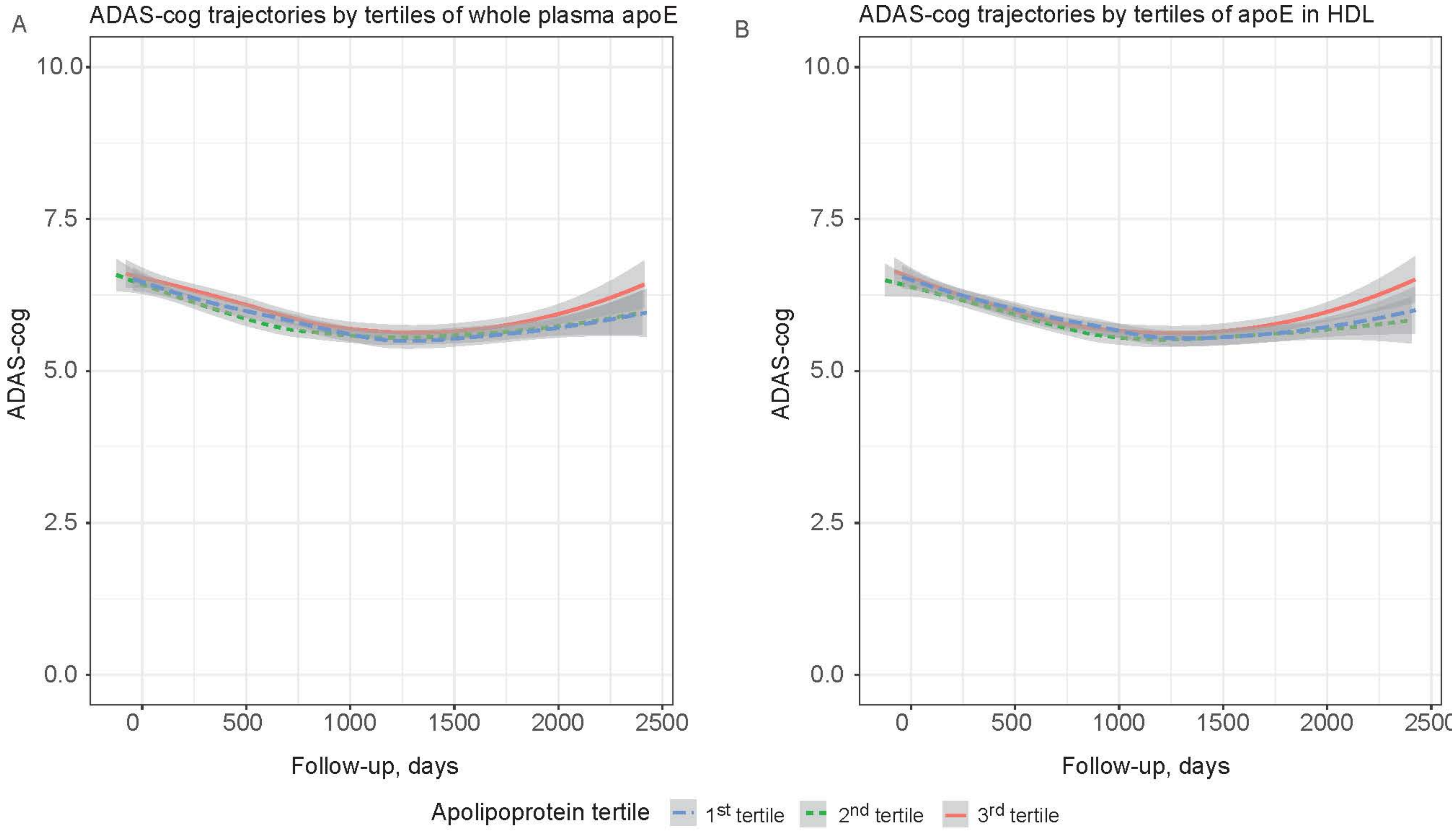

Abbreviations: ADAS-cog, Cognitive subscale of the Alzheimer Disease Assessment Scale. Apo, Apolipoprotein. HDL, High-density lipoprotein.

© 2020 Koch M et al. *JAMA Netw Open.*

eFigure 4. HDL and its apolipoprotein-defined subspecies and other apolipoproteins at study entry (2000 to 2002) and follow-up

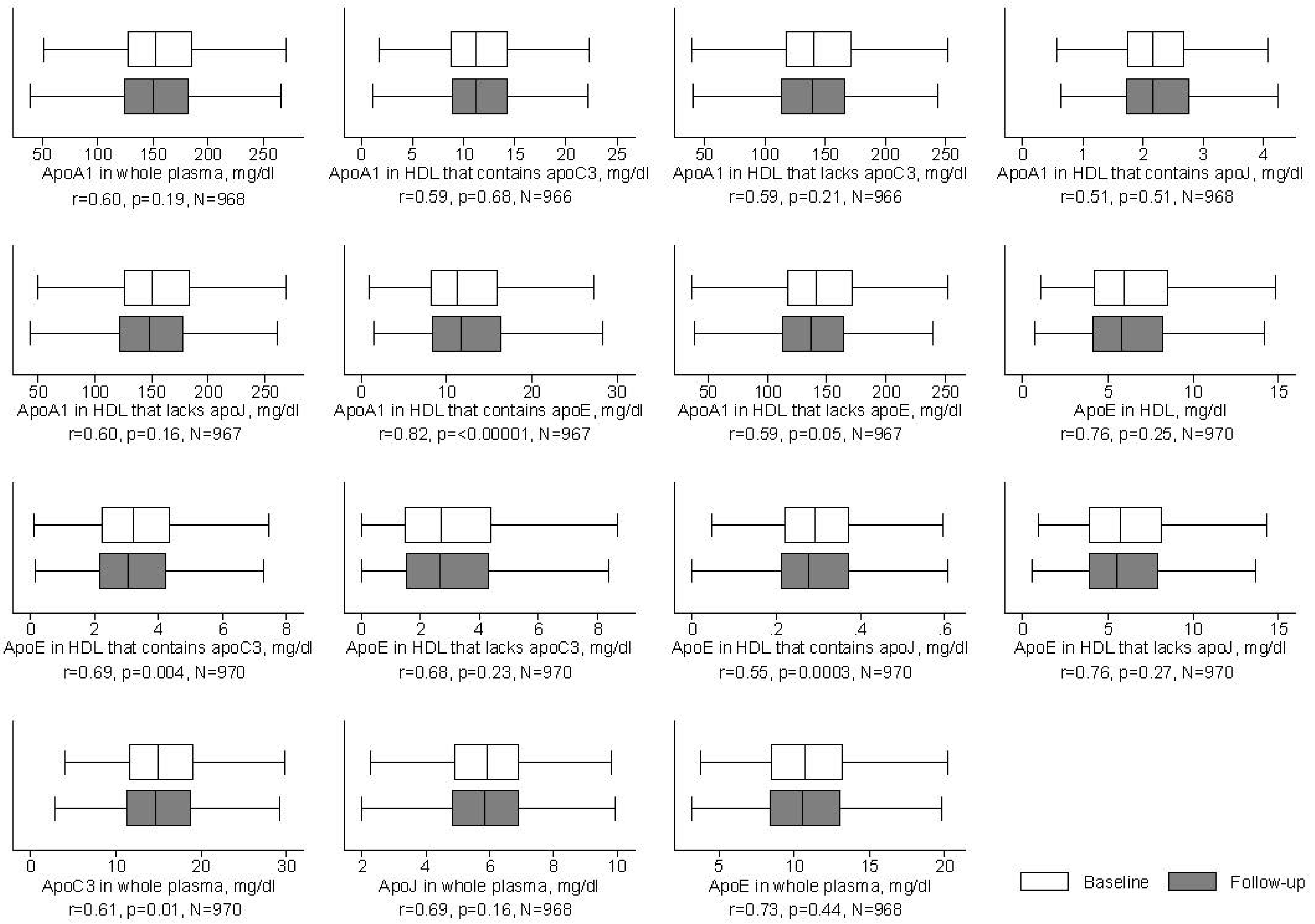

. Partial correlation coefficients (r) are shown of biomarkers assessed at study entry (2000 to 2002) and 3-year follow-up (4-year or 9-year follow-up for subset of participants) controlling for age at randomization and sex. P values for test of equality (p) are shown of biomarkers assessed at study entry and follow-up by using the Wilcoxon signed-ranks test.

© 2020 Koch M et al. *JAMA Netw Open.*
